# Latissimus Dorsi Transfer in Posterior Irreparable Rotator Cuff Tears

**DOI:** 10.2174/1874325001711010077

**Published:** 2017-02-28

**Authors:** Panagiotis P. Anastasopoulos, George Alexiadis, Sarantis Spyridonos, Emmanouil Fandridis

**Affiliations:** Hand Surgery-Upper Limb and Microsurgery Department, KAT General Hospital, Nikis 2 Str, Kifisia 145-61, Athens, Greece

**Keywords:** Irreparable rotator cuff tears, Latissimus dorsi, Massive rotator cuff tears, Shoulder surgery, Tendon, Transfer

## Abstract

**Background:**

Massive rotator cuff tears pose a difficult and complex challenge even for the experienced surgeon; inability to repair these tears by conventional means designates them as irreparable, while management becomes quite taxing. Several operative options have been suggested for the management of such lesions with varying degrees of success, while it is imperative to match patient demands and expectations to the predicted outcome.

**Methods:**

Research articles are examined and key concepts are discussed, in order to provide an evidence based review of the available literature. The anatomy and pathomechanics along with the indications, contraindications and surgical techniques are reported.

**Results:**

Transfer of the Latissimus dorsi has been used with success to restore shoulder function in deficits of the posterior rotator cuff. Although it can be used in a variety of settings, the ideal patient for a Latissimus dorsi tendon transfer is a young and active individual, with no glenohumeral osteoarthritis that has a severe disability and weakness related to an irreparable posterior cuff tear.

**Conclusion:**

Tendon transfers have proved to be a successful treatment option in salvaging this difficult problem, providing pain relief and restoring shoulder function. Despite the excellent functional outcomes and pain suppression following operation, a variety of factors may affect the outcome; thus making indications and preoperative assessment a valuable component.

## INTRODUCTION

Rotator cuff tears constitute a significant cause of pain and loss of upper limb function, an incident reflected in the increasing number of operations recorded per annum [1]. An estimated 10- 40% of all rotator cuff tears reported are massive [2-4] and may lead to even more severe impairment of shoulder function, as they are associated with persistent defects, weakness and poor outcomes [5]. The resulting symptomatology is deficit related; presenting in patients suffering from posterosuperior cuff tears as a decrease or loss of active external rotation and anterior elevation, resulting in spatial ataxia of the upper extremity [6].

A variety of classifications for massive rotator cuff tears has been proposed, although they are currently mostly defined the detachment of two or more tendons [7]. Several authors defined massive tears as those with an anteroposterior dimension greater than 5cm [8] whereas others have determined the size by measuring the amount of the exposed humeral head [9]. In addition massive tears may be characterized by their anatomic pattern to anterosuperior and posterosuperior [10]; the first concerns tears of the supraspinatus and subscapularis tendons, while the second is associated with tears of the supraspinatus and infraspinatus which may be combined with a compromised teres minor tendon.

Typically massive tears are chronic lesions characterized by gradual progression, and mostly affect older patients, while they only rarely occur as an acute injury secondary to trauma. Nevertheless chronic lesions may be complicated by acute injury and are often regarded as acute on chronic. Chronicity of these lesions may lead to muscle atrophy [11], tendon retraction [12-14] and fatty infiltration [15], posing a serious challenge even for the experienced shoulder surgeon in comparison to smaller tears; and despite of the technical difficulties encountered many of these are repairable [16].

Abnormal shoulder kinematics and joint forces consequent of a massive tear may conclusively lead to arthritic degeneration due to eccentric loading. Tears that cannot be repaired by conventional techniques on the rotator cuff footprint are deemed irreparable. Reported incidence ranges from 6.5%- 22.4% [17] and can be as high as 30% [18]. Reparability is affected by a variety of factors. An increased failure probability, has been correlated to an acromio-humeral distance of <7mm in a standard AP radiograph [3, 19] along with advanced fatty infiltration [19, 20] of the muscles above Goutallier stage 2 [15, 21]. Other factors that predispose to failure include the size of the defect and poor tendon quality [5, 22, 23].

A variety of treatment options has been proposed ranging from non-operative treatment to simple debridement, partial cuff repair with or without graft augmentation, tendon transfers and reverse total shoulder arthroplasty. Debridement with or without biceps tenotomy [24] has been proven to be effective, providing satisfactory functional outcomes and pain relief [25] despite the inability to restore strength [26]; nevertheless results may deteriorate with time and pain may reappear [24, 27]. Partial repair of the cuff on the other hand has been associated with the ability to restore normal function [28] providing strength improvement and satisfactory outcomes [29, 30]. Augmenting the tendons with allografts despite providing the ability to bridge the defect [31], has been associated with poor outcomes [32] and high failure rates [17, 33].

Reverse arthroplasty has been proven effective for older patients when significant joint degeneration is present. It provides the ability to restore active elevation [34-37], but in the setting of a posterior cuff deficiency it is not able to restore active external rotation; in which case it may be coupled with a latissimus dorsi (LD) tendon transfer to restore function [38-40]. Nevertheless it has been suggested recently in a study combining LD and teres major with reverse that it should be limited in patients with major disability and high grades of fatty infiltration, due to a high rate of complications [41].

Latissimus dorsi transfer has been first proposed in 1988 by Gerber for irreparable posterior cuff tears [23] as a means to restore active shoulder external rotation and has been used ever since with success [42-47].

The purpose of restoration of active external rotation has also been achieved by other tendon transfers such as the lower trapezius transfer to the infraspinatus tendon or insertion [48-50]; nonetheless LD still remains the most studied and reported transfer [23, 42, 51-55].

## PATHOMECHANICS

Normal shoulder kinematics and function is the result of the synergistic action of the muscles of the rotator cuff and the deltoid. These muscles act as a dynamic stabilizer by providing a centralizing force in the glenohumeral joint and allow for normal shoulder function [56]. The centralizing forces that are exerted into the glenohumeral joint act in couples and are present into the coronal and transverse planes [57]. The coronal plane balance is maintained by a force couple involving the deltoid and the inferior cuff, while balance in the transverse plane is maintained by the subscapularis anteriorly and infraspinatus-teres minor complex posteriorly [58-60]. In this setting any imbalance in the balancing force couples may result in abnormal shoulder kinematics and subsequently loss of function. Thus it is obvious that tears in the anterior cuff involving the subscapularis or posterior cuff involving the infraspinatus may result in uncoupling of the normal balance and produce abnormal forces and affect motion [57-60].

## ANATOMY-BIOMECHANICS

The latissimus dorsi is a triangular shaped muscle that originates from the posterior part of the iliac crest, the lumbar fascia, the T7-T12 vertebrae, the lower 3 or 4 ribs and several times with a few muscle fibers from the inferior scapular angle; and is inserted into the floor of the bicipital groove of the humerus. It is innervated by the thoracodorsal nerve, and its original function is extension, adduction and internal rotation of the arm [61].

The purpose behind LD tendon transfer is to restore active shoulder external rotation and normal shoulder function in the setting of an imbalance caused by a posterior cuff deficient force couple [62]. The hypothesis behind restoration of external rotation following LD tendon transfer was supported as early as 1936 by L’Episcopo [63] who used it for obstetrical plexus paralysis, followed by Gerber *et al*. who successfully used it to treat massive and irreparable RC tears [23, 42]. Transfer of the LD provides the ability in the context of a massive irreparable posterior cuff tear, to bridge the defect and provide an external rotation force thus restoring the force couple and improve shoulder motion [64]. With the transfer procedure the function of LD changes from an internal rotator to an external rotator [61]; this is achieved by either a true muscular activity or a tenodesis effect exerting a passive force that balances the force couple [65].

Muscular activity has been recorded in several EMG studies following transfer procedures, and has been exhibited that LD activates synergistically and contributes besides external rotation to both adduction [66] and abduction [67, 68]. Furthermore the LD tendon acts as a depressor of humerus resisting cranial migration, which in turn allows for more effective deltoid function during abduction and forward flexion [55, 65]. Nevertheless, despite the marked functional improvement reported with LD transfers in posterior irreparable tears, the normal rotator cuff function cannot be fully restored; owing mostly to abnormal forces exerted on the glenohumeral joint relative to the inferior LD anatomic position in regards to infraspinatus and teres minor [62].

## INDICATIONS-CONTRAINDICATION FOR LD TRANSFER

In the absence of a perfect solution, treatment of massive irreparable posterior RC lesions has proven to be quite challenging, adding to the surgeon’s dilemma regarding the choice of patient and treatment option. Tendon transfers have proven to be a valuable asset in restoring function and improving outcomes in the setting of an irreparable cuff tear. However a multitude of elements may influence the outcome and effectiveness of such operations, limiting their use to selected patients; and rendering the use of indications and contraindications, an essential component (Table **1**).

Preoperative assessment must take into consideration a variety of factors, including age, pain, disability, functional demands and patient expectations; additionally the patient must be educated on anticipated functional outcomes. Other factors that may affect operative outcomes must be considered as well, such as medical comorbidities, subscapularis and deltoid function, coraco-acromial arch integrity the presence of arthritic changes and the degree to which a patient is obliged and will comply with extended rehabilitation.

The principal complaints on presentation in patients with symptomatic massive RC tears are usually pain, weakness and loss of motion. The cause of pain must be determined during physical examination and distinguished from other causes of shoulder pathology [69]. Massive irreparable RC tears are common causes of severe pain and are often extremely disabling minimizing in this context the choice on other treatment modalities.

Typically patients with posterosuperior cuff tears present with reduced forward flexion and abduction and are unable to actively externally rotate the arm [70, 71]. A positive external rotation lag sign and a positive hornblower’s sign are indicative of posterior cuff deficits and external rotation inability [72, 73]. Moreover a positive hornblower’s sign has been associated with irreparable teres minor tears [73] and advanced fatty infiltration [7, 14] and is related to poor outcomes in tendon transfers [74]. This has been highlighted in the studies of Gerber *et al*. [55] and Costouros *et al*. [74] who reported lower outcome scores and decreased external rotation in patients with advanced fatty degeneration. However Nové-Josserand *et al*. found that patients with severe atrophy had greater increments in functional outcome scores than patients with moderate atrophy or without atrophy and bigger gains in active external rotation during abduction; suggesting that the transferred LD compensates the deficient teres minor [75]. The positive influence an intact teres minor may have on LD tendon transfer may be partially explained by the fact that it has a greater excursion in comparison to the other rotator cuff muscles; not impending thereby the excursion of the LD [74, 76].

Weakness may present with varying degrees ultimately leading to pseudoparalysis; referring to the inability of active external rotation or forward flexion in the absence of nerve lesions or unhindered passive motion. It has been suggested that pseudoparalysis is a contraindication to LD transfer [77], and it is related to poor outcomes [65]; as it does not provide the required strength to overcome a pseudoparalytic shoulder and achieve elevation not present preoperatively [65]. However recent evidence suggests that in patients with an intact subscapularis it may not be a contraindication as it may provide good functional results in that particular group, although further studies may be required to provide additional data for that matter [78]. Nevertheless despite the fact that there is no consensus regarding the degrees of anteflexion for a shoulder to be considered pseudoparalytic, in a recent study [79] patients with mobility lower than 80° exhibited bigger increments in anteflexion than patients with greater range of motion; suggesting that these patients still gain from LD tendon transfers. A finding confirmed by a study by Nové-Josserand *et al*. who reported on five pseudoparalytic patients that gained mobility postoperatively and overcame the pseudoparalytic state [75].

Furthermore patient observation may reveal the status of the periscapular muscles, revealing atrophies and possible deficiencies as the detachment of deltoid muscle origin. Subscapularis and deltoid muscles must be intact for a successful LD transfer as deficiencies may cause imbalances in the force couples subsequently causing instabilities in both planes [47]. Poor outcomes have been reported by many authors for LD transfer in patients with subscapularis tears, rendering it a definitive contraindication [23, 43, 44, 46, 61, 80]. However several studies reported good results with no impact on final outcomes following repair of partial upper third subscapularis tears [47, 65, 75, 79, 81].

Imaging studies play a critical role in preoperative assessment and evaluation of the defects. Plain film radiographs are useful in determining the morphology and status of the glenohumeral joint. Osteoarthritic changes of the glenohumeral joint are evident in simple radiographs and the degree of glenoid erosion can be easily assessed [82, 83]. The presence of severe osteoarthritis is coupled with inferior outcomes in the setting of LD tendon transfer [84] while furthermore in patients of young age that are not candidates for a total shoulder arthroplasty, LD transfer may potentially restore function and slow arthritic progression [55]. A decreased acromiohumeral distance <7 mm may also be exhibited which is correlated to the size of the RC tear, tendon irreparability and varying degrees of fatty infiltration [85]. Typically an acromiohumeral distance <7mm is associated with superior migration of the humeral head and indicates tears of the infraspinatus that mainly acts in lowering the humeral head [86-89]. The integrity of the coracoacromial arch may be assessed as well, an important structure that stabilizes anterosuperior movement when there is a tear of the supraspinatus and any other tendon [18, 90, 91]. This is due to the loss of joint compressive forces in the setting of a massive irreparable tear, that are unable to be compensated by the remaining muscles; leading to an imbalance between a deficient rotator cuff and a strong deltoid with subsequent anterosuperior subluxation [92, 93]. In this context an intact arch remains the only passive restraint against superior subluxation, which when severely compromised will have an impact on overhead function [94].

Computed tomography scans were successfully used to evaluate the degree of rotator cuff atrophy and the degree of fatty infiltration, although MRI studies are more effective in the study of soft tissue deficits and are used more often. Muscle belly atrophy can be determined effectively [95] and is correlated to poor functional outcomes following repair [96]. The size of the defect can be easily assessed in MRI studies, as well as tendon quality [5, 22, 23] two factors that directly affect the reparability of a tear. Tendon retraction [97] can be evaluated as well, along with muscular fatty infiltration, an indicator of chronicity that is coupled to the probability of a failed repair when above Goutallier stage 2 [15, 96].

Ultrasonographic reports of soft tissue deficiencies seem to correlate with MRI findings in up to 85% of the cases [98, 99] although the outcome and repeatability is directly related to the experience of the technician.

## SURGICAL TECHNIQUES

Latissimus dorsi tendon transfer has become a valuable choice for reconstruction of irreparable posterosuperior rotator cuff tears. Since its inception, several modifications of the original technique have been suggested, while other considerations have risen such as the method and site of fixation. Variable means of fixation have been used by several authors such as transosseous sutures [23, 47], classic anchors [45, 81, 100] and interference screw [101]. A common mode of repair failure has been discussed by several authors [102, 103] and involves splitting of the transferred tendon by sutures mainly due to its reduced thickness [104]. A recent biomechanical study supports this finding by reporting that the weakest element was the tendon itself and not the anchor – suture construct [103]; while they conclude that interference screw fixation presents higher or similar biomechanical properties to anchor fixation whereas tubularization of the tendon which is performed in conjunction, may address the reduced thickness variable [103]. Regarding this matter, other authors suggested harvesting the tendon with a small bone chip may help alleviate this issue [54].

Several authors agree with the hypothesis, that lower thickness of the transferred tendon is responsible for secondary failures [75, 84, 105, 106]. The incidence of late LD tendon rupture has been reported to occur in 20-30% of the cases while it has been suggested that augmentation may help to overcome this issue [18]. A number of materials have been used for enhancing LD structural integrity at the insertion point including, synthetic biomaterials autografts and allografts. Vicryl mesh [84] has been used with success suggesting that the material provides strong fixation points at the insertion site [84]. Absorbable PDS reinforcement [75] has also been recommended, suggesting an enhanced tenodesis effect due to the ability for the transplant to be placed under tension; coming to agreement with Aoki *et al*. regarding augmentation with a Teflon felt [107]. Other authors reported the use of Achilles tendon or double looped semitendinosus allografts, which in addition to reinforcing the insertion site they may allow for lengthening of the tendon if required [108]. Further options include tensor fascia lata [106] and teres major augmentation.

The combined use of LD and teres major has been studied extensively. It has been used initially by L’Episcopo for restoring external rotation secondary to obstetric brachial plexus paralysis and later by Hoffer [109]. The role in massive irreparable cuff tears is providing tissue for coverage and enhancing strength of the tendon complex [110]. Isolated LD transfers versus combined LD and teres major transfers have been reported to have lower gains in strength postoperatively [72, 110]. Biomechanical studies have shown that isolated teres major transfers are more efficient in restoring normal shoulder function versus isolated LD transfers or a combination of them [67, 111]. Nevertheless it has been demonstrated [110] that the use of isolated LD transfer provides better external rotation and forward flexion, while joint degeneration progresses slower and patient satisfaction remains high [43-45, 47, 65, 81, 112]. Moreover isolated teres major transfer remains difficult to perform due to a short excursion [76, 113].

The site of tendon fixation remains controversial. Several authors support attachment to the superior facet of the greater tuberosity or the subscapularis or the stump of the supraspinatus in order to obtain a more anterior fixation [45, 65, 68, 69, 76, 77, 111]; gaining thereby a supplementary tenodesis effect. Other authors [114, 115] recommended fixing the tendon at the infraspinatus insertion site on the middle or inferior facet in order to achieve better external rotation. Furthermore a recent biomechanical finite element study suggested that the best option for restoration of external rotation and anterior elevation is fixation at the insertion of the infraspinatus tendon [67, 111]. In regards to operative technique, LD transfer may be accomplished either by open technique, or in combination with arthroscopy.

## SINGLE INCISION

The opportunity to perform the LD tendon transfer via a single incision thereby avoiding deltoid injury, was made possible by translating the site of fixation posteriorly and inferiorly [45, 75]. In this context Habermayer *et al*. [45] suggested a single incision axillary approach and reported comparable clinical results to the standard double incision [23, 42, 81, 116]. Boileau *et al*. [115] used a modification of the deltopectoral approach described by L’Episcopo. The single incision according to Habermayer [45] is performed in a lateral decubitus position. Exposure is achieved through a triangular incision from the lateral border of the scapula to the inferior scapular angle and from the axillary pouch to its apex. Identification and separation of the LD from the teres major follows. Detachment of the tendon occurs. The infraspinatus is approached through the plane between deltoid and teres minor. The arm in 90° of abduction and external rotation helps identify the greater tuberosity. The tendon is then inserted and attached at the site of the infraspinatus insertion.

## DOUBLE INCISION

**Fig. (1):**
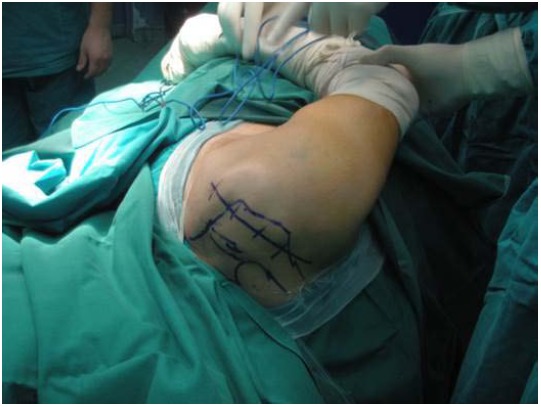
Superior approach with an incision 1 cm medial to the lateral border of the acromion.

**Fig. (2):**
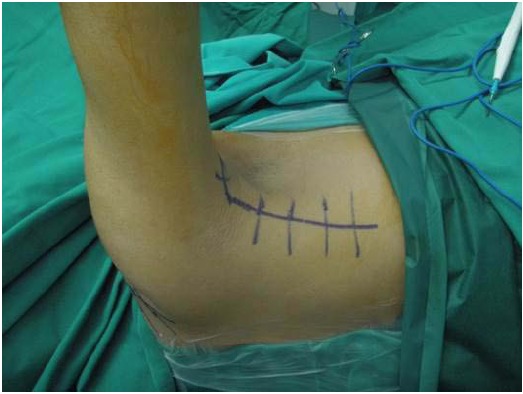
Axillary approach on the lateral border of the latissimus dorsi.

**Fig. (3):**
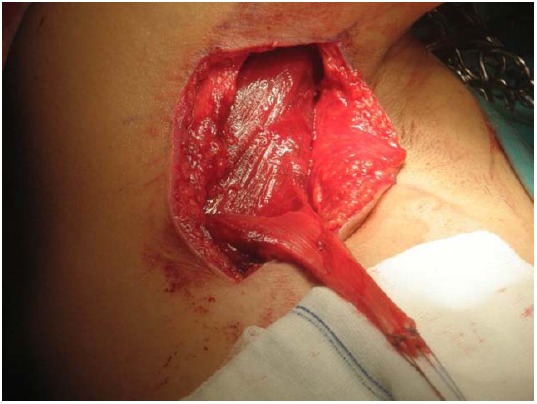
Dissection of the pedicle and separation of the latissimus dorsi tendon.

**Fig. (4):**
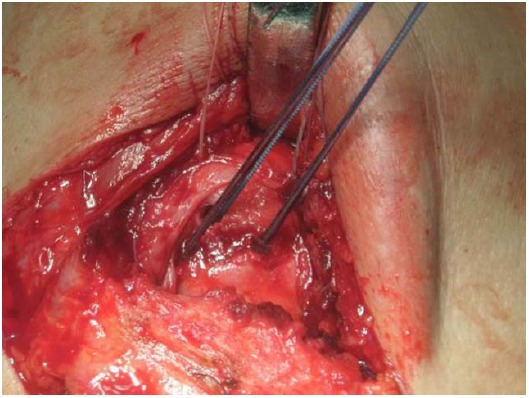
Preparation of the denuded supraspinatus footprint for fixation of latissimus dorsi with anchors.

The original technique suggested by Gerber *et al*. in 1988 [23] involved a double incision, *via* the axilla and superiorly via the deltoid [42]. This technique has been popularized by other authors and reported good results in the absence of a subscapularis tear [47, 81, 117]. The operation is performed in a lateral decubitus position. A superior approach is performed with an incision lateral to the acromioclavicular joint, allowing for exposure of the rotator cuff through a deltoid split (Fig. **1**). The axillary approach is performed through an incision on the lateral border of the LD (Fig. **2**). The muscle is released from the humerus and separated from teres major (Fig. **3**). Following exploration of the neurovascular bundle the muscle is transferred through the interval between the deltoid and infraspinatus and teres minor. Attachment of the tendon follows at the supraspinatus insertion with anchors while attachment of the remaining rotator cuff to the medial edge of the muscle is performed (Fig. **4**) [62]. In addition a more anterior fixation may be achieved with suturing on the superior border of the subscapularis.

## ARTHROSCOPICALLY ASSISTED

**Fig. (5):**
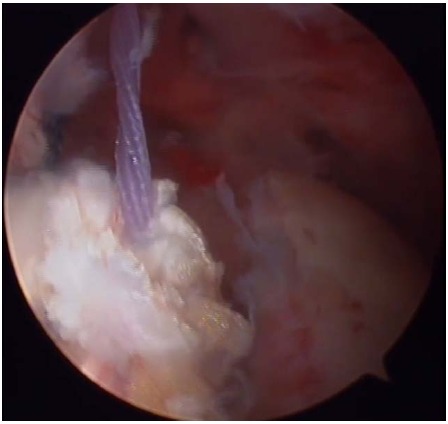
Transfer of the latissimus dorsi tendon by pulling the sutures through an anterior portal.

**Fig. (6):**
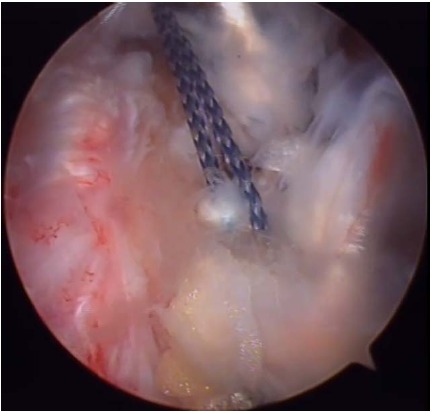
Anchor fixation of the latissimus dorsi over the supraspinatus footprint.

Arthroscopically assisted techniques are technically demanding procedures, while they permit for reduced muscle damage in contrast to open surgery. Injury of the deltoid muscle may occur during open transfer [118] an incident that is related to poor clinical outcomes [10, 43, 65, 74], while it has been exhibited that following open surgery it is unable to regain strength [61]. Therefore arthroscopically assisted procedures may potentially help avoid such issues. The procedure typically involves dissection and preparation of the LD through an axillary approach while the glenohumeral joint is initially inspected via arthroscopy through a posterior portal [108]. The insertion site is prepared arthroscopically by decorticating the infraspinatus and supraspinatus footprint. A subdeltoid tunnel is created by blunt dissection through the interval of the deltoid- teres minor. The tendon is then passed under the acromion by pulling the sutures through the anterior portal (Fig **5**). Fixation of the tendon follows with the suture anchors and once secured the anterior edge is fixed to the subscapularis (Fig **6**). 

Recently a mini-open approach has been introduced for LD harvesting to be used in combination with arthroscopy for transfer and fixation [101]. A small axillary incision around 5 cm for preparation of the LD is achieved with the use of an ultrasound Doppler. Preoperative identification of the pedicle using ultrasonography, allows for accurate placement of the incision and straightforward dissection while minimizing incision length. Fixation of the tendon follows by means of arthroscopy.

## REVERSE SHOULDER ARTHROPLASTY AND LD TRANSFER

Massive rotator cuff tears are frequently linked with superior humeral head migration [119-122] which without appropriate treatment may lead to degenerative changes of the glenohumeral joint. Cuff tear arthropathy usually presents with loss of active elevation, although a subset of patients may present with severe dysfunction due to a combined loss of elevation and external rotation [38]. Reverse shoulder arthroplasty has been used with success in treating cuff tear arthropathies and restore active elevation [34-37]; it does not however restore active external rotation and may lead to reduced functional results [34, 123, 124]. Pseudoparalysis of external rotation has been mainly attributed to deficiency of the teres minor [72, 73, 125], relative to chronicity with ensuing atrophy or fatty infiltration [7, 126]. In this context, extending indications of LD tendon transfer to be performed in conjunction with reverse shoulder arthroplasty has provided a solution; as it has been demonstrated to increase external rotation and provide a better control of the arm in space [38-40].

## OUTCOMES

Latissimus dorsi tendon transfers have been associated with satisfactory results [43, 45, 55, 65, 75, 81, 84, 105]. Table **2** Reported pain, range of motion and function have been shown to improve postoperatively [43, 45, 55, 65, 75, 81, 84, 105]. Patient satisfaction following operation remains high [32, 43-45, 47, 81, 112], while a high percentage of patients has been reported to be willing to undergo the operation again [42, 78, 84]. Operative outcomes have been demonstrated to be sustained in the long term, preserving pain relief and range of motion [55, 127]. There are some reports however, demonstrating deterioration of results with time and reemergence of pain [24, 27]. Recently Gerber *et al*. [55] associated poor outcomes following operation, with a large critical shoulder angle [128]. They noted significantly better outcomes in the low angle group (≤36°) in regards to flexion, abduction and mean relative constant score; while abduction strength and external rotation were comparable. These findings were attributed to better stability in abduction and flexion, theoretically resulting from a deltoid force vector that is centered in the glenoid in shoulders with a low critical angle [55]. Thus they suggested correction of a large critical angle >36° to be taken into consideration.

Despite the important improvements noted on range of motion and pain relief, strength gains following the tendon transfer are low. Gerber *et al*. [55] noted an approximately 40% strength gain in women and 25% in men in relation to preoperative levels. In a recent systematic review which included 10 studies the authors indicated that a 70% gain in abduction strength can be expected [64]. Possible causes of low strength gains suggested include, the inability of LD to center the humeral head [61]. The noted strength gains however, do not seem to influence daily living activities [61]. Furthermore another factor that may influence postoperative outcomes is the compliance and adherence of the patient to a strict physiotherapy regime, that is required in order to develop the neuromuscular control to recruit the LD as an external rotator instead of an internal rotator [129].

Joint degenerative changes has been recorded in several studies and results were quite controversial. Gerber *et al*. [55] noted structural deterioration comparable to non-operative management, although progression was reported slower. The described osteoarthritic changes were commonly restricted in 1 or 2 stages of the Hamada classification [82], while another group [130] reported significant osteoarthritic changes with time. However, it has been reported that radiologic findings regarding arthritic degeneration are not related to the clinical outcomes [43, 54, 117]. Nevertheless joint degeneration is anticipated [43, 75, 117] due to degradation of the tenodesis effect over time [131], a finding evident in several studies that demonstrated a decrease in acromiohumeral distance in the mid [130] and long term [55, 127] in comparison to the initial postoperative outcome. It has been suggested however, that potentially LD tendon transfer may prolong the time till arthroplasty [131].

Primary operations have been reported to obtain better results than revision operations [43, 44, 47, 54, 75, 79, 84, 117]. In addition several investigators noted better outcomes in younger patients [127]. Arthroscopically assisted techniques have been reported to obtain results comparable to the open transfers [103, 108]. Moreover a recent clinical study reported significant improvements regarding strength and functional outcome scores in patients treated with arthroscopically assisted LD tendon transfer in comparison to patients treated with arthroscopic partial repair [132]. Regarding the approach, there are no studies in the literature with strong statistical data to provide a valid comparison between single and double incision; although it has been reported that results from single incision are comparable to the standard double incision technique [81, 116]. Complications reported include, the formation of hematoma [84, 105, 117] in up to 14,3% of the patients, and the appearance of infection [61, 84, 105, 117] in up to 7,7% of the patients. Late transferred tendon rupture [44, 47, 54, 80, 105, 117] has been recorded ranging from 5,5% up to 44%. Nerve palsies following operation have been reported as well although in most cases were transient [43, 54, 117].

## CONCLUSION

Latissimus dorsi tendon transfer for the treatment of irreparable posterior cuff tears is a demanding and complex procedure that may restore shoulder function and provide pain relief. In addition it may help restore strength to some extent, but this improvement should not be expected to be significant. Outcome results may vary and directly rely on careful patient selection while underlying pathology must be taken into consideration. Patients should be able to withstand and comply with long rehabilitation protocols, as well as be informed on the benefits expected following operation. In this context patient expectations should match anticipated results.

## Figures and Tables

**Table 1:** Indications, contraindications and complications of latissimus dorsi tendon transfer.

Indications	Contraindications	Complications
Patients • Young age, active individuals • Older patients with increased functional • demands, bodily active Clinical • Severe functional disability due to • Weakness and loss of motion • Severe pain caused by a massive • Irreparable cuff tear Compliance • Patients that will comply with long •rehabilitation Imaging and intraoperative • Fatty infiltration – retraction • Not amenable to repair	Absolute	• Postoperative hematoma • Infection • Late / secondary tendon rupture • Anchor pull out • Nerve palsies
	• Advanced age • Comorbidities (infection *etc*) • Severe joint degeneration (Hamada ≥ stage 2, Samilson ≥ stage 1) • Compromised Coracoacromial arch • Anterosuperior subluxation • Subscapularis deficiency – atrophy – fatty degeneration • Deltoid deficiency – detachment • Nerve injuries • Inability to comply with long rehabilitation	
	Relative	
	• Subscapularis partial tears • Advanced fatty degeneration of the teres minor • Acromio- humeral distance < 7 mm • Moderate joint degeneration (Hamada – Stage 1-2) • Preoperative weakness - pseudoparalysis	

**Table 2:** Functional outcome scores following LD tendon transfer (CS: Constant Score, SSV: Subjective Shoulder Value, SSI: Shoulder Score Index).

Authors	Journal	Year	No. of patients	Technique	Age	Follow up	Preoperative functional measurements	Postoperative functional measurements
El-Azab *et al*.	J bone Joint Surg Am	2015	93	Gerber	56 years (40-72)	111,6 months (79,2-132)	44% relative CS 30 ASES score	71% relative CS 70 ASES score
De casa *et al*.	J Orth Surg Res	2014	14	Arthroscopy assisted	59 years (52-66)	52 months (36-77)	33 CS	59 CS
Castricini *et al*.	J bone Joint Surg Am	2014	27	Arthroscopy assisted	60 years (46-67)	27 months (24-36)	36 CS	74 CS
Villacis *et al*.	Arthrosc Tech	2013	8	Arthroscopy assisted	54 years (49- 60)	Mean 14 months	63 SSI	70 SSI
Gerber *et al*.	J bone Joint Surg Am	2013	45	Gerber	56 years (37-67)	146.6 months (122-184)	47,3 CS 29% SSV	63,8 CS 70,1% SSV
Debeer *et al*.	Acta Orthop Belg	2010	26	Gerber	56,5 years (42-66)	43,3 months (13-124)	39 CS	60 CS
Weening *et al*.	Int Orthop	2010	16	Gerber	60 years (49-71)	26 months (7-73)	32,5 CS	50,3 CS
Moursy *et al*.	J bone Joint Surg Am	2009	42	Gerber (n =22) Bone chip (n=20)	58 years (50-75)	Mean 47 months	43,4 (Gerber technique) 40,2 (bone chip)	64,8 ASES score 74,2 ASES score
Nové – Josserand *et al*.	Orthop Traumatol Surg Res	2009	26	Gerber	55,5 years (36-71)	34 months (24-62)	50 CS	74 CS
Habermayer *et al*.	J bone Joint Surg Br	2006	14	Single incision	61 years (47- 76)	32 months (19-42)	46,5 CS	74,6 CS
Gerber *et al*.	J bone Joint Surg Am	2006	69	Gerber	61 years (49- 72)	59 months (24 – 126)	55% CS	73% CS
Ianotti *et al*.	J bone Joint Surg Am	2006	20	Gerber	54,8 years (44-68)	39 months (24- 85)	40 PENN score	66 PENN score
Miniaci *et al*.	J bone Joint Surg Am	1999	17	Gerber	55 years (32-77)	51 months (24-72)	6,8 UCLA score	16,4 UCLA score
Aoki *et al*.	J bone Joint Surg Br	1996	12	Gerber	64 years (48- 82)	35,6 months (26-42)	11,8 UCLA score	28 UCLA score

## LIST OF ABBREVIATIONS

AP: = Anteroposterior
ASES: = American Shoulder and Elbow Surgeons
CS: = Constant Score
EMG: = Electromyographic
LD: = Latissimus Dorsi
MRI: = Magnetic Resonance Imaging
PDS: = Polydioxanone Sutures
RC: = Rotator Cuff
SSI: = Shoulder Score Index
SSV: = Subjective Shoulder Value
UCLA: = University of California at Los Angeles
